# Study protocol for evaluating integrated urban development plans: impacts on health, social equity, and environmental sustainability in Germany

**DOI:** 10.1186/s12889-026-28247-7

**Published:** 2026-06-24

**Authors:** Justus Tönnies, Aline Krumreihn, Franziska Stelzer, Ellen Senck, Sabine Bongers-Römer, Imke Stalling, Katharina Gröne, Karin Bammann, Heike Köckler, Gabriele Bolte

**Affiliations:** 1 Department of Social Epidemiology, University of Bremen, Institute of Public Health and Nursing Research, Bremen, Germany; 2 University of Bremen, Health Sciences Bremen, Bremen, Germany; 3 Bochum University of Applied Sciences, Bochum, Germany; 4 Wuppertal Institute for Climate, Environment and Energy, Wuppertal, Germany

**Keywords:** Urban development, Health equity, Complex intervention, Process evaluation, Outcome evaluation, Mixed-methods study, Environmental justice, Social justice, Ecological sustainability

## Abstract

**Background:**

The urban environment plays a significant role in shaping residents’ health, wellbeing, and opportunities for social participation. Urban development interventions have been shown to positively influence physical activity, mental health, quality of life, and self-reported health status. However, most previous studies have focused on single measures or specific environmental aspects. Integrated Urban Development Plans (IUDPs), by contrast, combine interventions from multiple domains, such as housing, infrastructure, green spaces, and social participation, to promote health equity and sustainable urban transformation. The SalusTransform project aims to evaluate IUDPs in three German cities regarding their implementation processes as well as their impacts on health, social equity, and ecological sustainability.

**Methods:**

We will compare three IUDP intervention areas with three control areas in the respective cities using a mixed-methods design. Quantitative and qualitative data will be collected to assess both process and outcome quality of the IUDPs. Primary data collections include resident surveys and focus groups, qualitative interviews with stakeholders, as well as environmental and infrastructural assessments, including air-quality measurements, site visits, and observations related to active mobility and urban green spaces. Secondary data on population structure, aggregated health indicators, and local implementation processes will be provided by the municipalities. Analyses will follow a difference-in-differences approach to identify changes attributable to IUDP implementation while accounting for similar developments in the control areas.

**Discussion:**

By building a comprehensive database that links health-related, social, and environmental indicators, SalusTransform will provide valuable evidence on the real-world effects of IUDPs. Findings will inform municipalities, policymakers, and practitioners about the effectiveness and transferability of IUDPs and contribute to the institutionalisation of their systematic evaluation. Close collaboration with local authorities will ensure continuous monitoring of changes and strengthen the practical relevance of the research. Targeted communication strategies will be implemented to engage population groups that are often underrepresented in research and urban planning processes.

**Trial registration:**

This study was prospectively registered with the German Clinical Trials Register (DRKS00036042) on May 28th, 2025.

**Supplementary Information:**

The online version contains supplementary material available at 10.1186/s12889-026-28247-7.

## Background

The urban environment plays a significant role in shaping health and wellbeing. With an estimated 75% of the global population expected to live in cities by 2050, urban development and urban planning are becoming more and more critical topics for addressing complex public health challenges [1, 2]. Cities concentrate both opportunities and risks: while they may provide access to healthcare, education, employment, and cultural resources, they are also sites where air pollution, limited green space, traffic-related hazards, and social stressors converge [3, 4]. Due to their diverse socioeconomic structures, in cities health inequities are particularly present and need to be considered when urban development measures are conceptualised and implemented [5].

Urban development interventions especially aim to address problems in disadvantaged neighbourhoods. Existing research has demonstrated that urban development interventions can have positive effects on residents’ health and well-being. Studies and reviews have reported improvements in outcomes such as physical activity, mental health, and perceived health status. These positive effects followed interventions targeting specific aspects of the built or social environment. Examples include the external and internal upgrading of private homes, the improvement of (path) infrastructure, and the redesign of public (green) spaces. Such measures were often complemented by interventions aimed at social interaction, such as offering shared neighbourhood activities or establishing local political bodies to involve residents [6–12]. However, most studies have focused on single or narrowly defined measures rather than comprehensive, multi-component strategies. Evidence on the combined or synergistic effects of integrated urban interventions – comprehensively addressing dimensions of the natural, built and social environment at the same time – remains limited.

To combine and integrate various types of interventions while considering the interconnectedness of different domains of action and the need for cross-sectoral collaboration, Integrated Urban Development Plans (IUDPs) are developed in Germany [13]. As informal, integrative instruments of urban development, IUDPs play a particularly important role in improving urban living conditions in neighbourhoods facing multiple challenges. Based on preparatory analyses, municipalities first identify sub-areas that are structurally, economically, and socially disadvantaged and derive thematic priorities accordingly. These priorities encompass health-relevant determinants such as access to green and public spaces, barrier-free infrastructure, or healthcare provision. Although IUDPs are always developed for a specific urban context and involve various municipal departments and public participation for concept development, they follow a comparable logic and often employ similar interventions. IUDPs describe both a process and a bundle of measures through which various stakeholders aim to steer the development of a city or district in a desired direction. They serve as overarching strategies that initiate additional activities and measures in a given urban area, even when financed through separate funding sources. With their diverse urban renewal measures, IUDPs are an example of complex interventions implemented in an urban setting [14–16]. They meet the criteria for complex interventions as defined by Skivington et al. [17].

Up to now, there is a lack of comprehensive evaluations of IUDPs that analyse real-world impacts on health, equity, and sustainability of neighbourhoods. Thus, the project SalusTransform will include both process and outcome evaluations and aims to assess how IUDPs are implemented and whether they improve the social determinants of health of the built, natural and social neighbourhood environment as well as residents’ quality of life, to promote greater health equity. We will collect quantitative and qualitative data using a mixed methods approach and employ participatory data collection methods. We will not implement any interventions by ourselves.

## Methods

### Study setting

**Fig. 1:**
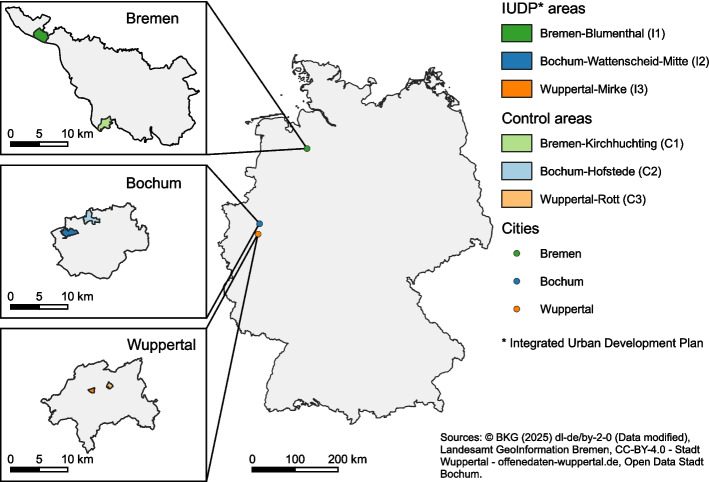
Localisation of the six study areas within Germany and the respective cities

This study will evaluate the impact and process quality of IUDPs in three urban areas in Germany. The intervention areas are the districts of Blumenthal (I1) in the city of Bremen, federal state of Bremen in northern Germany, Wattenscheid-Mitte (I2) in Bochum and Mirke (I3) in Wuppertal, both cities located in the federal state of North Rhine-Westphalia in the western part of Germany. In each city, a further district will serve as control area in which no IUDP will be implemented. These control areas are Bremen Kirchhuchting (C1), Bochum Hofstede (C2) and Wuppertal Rott (C3). For the localisation of all six study areas within Germany and the respective cities, see Fig. 1.

The study protocol is reported and implemented according to the Standard Protocol Items: Recommendations for Interventional Trials (SPIRIT) Statement (see Additional file 1 for the checklist).

### Description of study areas and IUDPs

The three intervention areas and the three control areas are located in three different cities in Germany, with populations ranging from 360,000 (Wuppertal) to 585,000 (Bremen) inhabitants [18]. All study areas have a similar population structure across various social indicators and face comparable challenges concerning population characteristics, infrastructure and environmental conditions. Further details about the population characteristics of the six study areas are presented in Table 1.

**Table 1:** Population characteristics of the intervention and control areas

	**Bremen Blumenthal**^**a**^	**Bremen Kirchhuchting**^**b**^	**Bremen** **total**	**Bochum** **Wattenscheid**^**a**^	**Bochum Hofstede**^**b**^	**Bochum total**	**Wuppertal Mirke**^**a**^	**Wuppertal Rott**^**b**^	**Wuppertal total**
Population	10,706	9,356	586,271	23,309	10,341	375,204	8,573	10,188	358,200
Proportion of migrants^c^	57.1%	61.9%	43.5%	46.0%	44.4%	34.9%	59.9%	52.2%	42.6%
Proportion of foreigners^d^	34.7%	32.3%	22.9%	27.2%	22.6%	17.0%	38.2%	31.5%	23.0%
Unemployment rate	22.0%	19.0%	12.6%	12.2%	9.5%	7.4%	10.8%	10.0%	10.9%
Proportion of unemployment assistance^e^	29.7%	25.9%	16.2%	25.8%	18.2%	14.1%	24.0%	23.9%	15.5%
Age structure									
≥ 65 years	16.5%	18.4%	20.6%	26.4%^f^	26.6%^f^	29.3%^f^	11.8%	16.0%	20.6%
< 18 years	23.6%	22.5%	17.1%	17.9%	22.3%	15.2%	18.9%	19.5%	14.1%
Old-age dependency ratio^g^	27.6%	31.1%	33.1%	n. a	n. a	n. a	14.6%	19.0%	35.2%
Youth dependency ratio^h^	39.4%	38.1%	27.5%	n. a	n. a	n. a	27.0%	30.2%	33.7%

^a^Intervention area

^b^Control area

^c^Migrants: Non-German nationals and naturalized Germans, and German children < 18 with at least one parent with a migration background

^d^Foreigners (% of migrants): Individuals without German citizenship

^e^Proportion of residents under 65 receiving unemployment benefits (basic income support)

^f^Owing to methodological differences in data collection in Bochum, this age group includes individuals aged ≥ 60 years rather than ≥ 65 years

^g^number of people aged 65 + divided by the working-age population (15–64), multiplied by 100

^h^number of people aged 0–14 divided by the working-age population (15–64), multiplied by 100

**Fig. 2:**
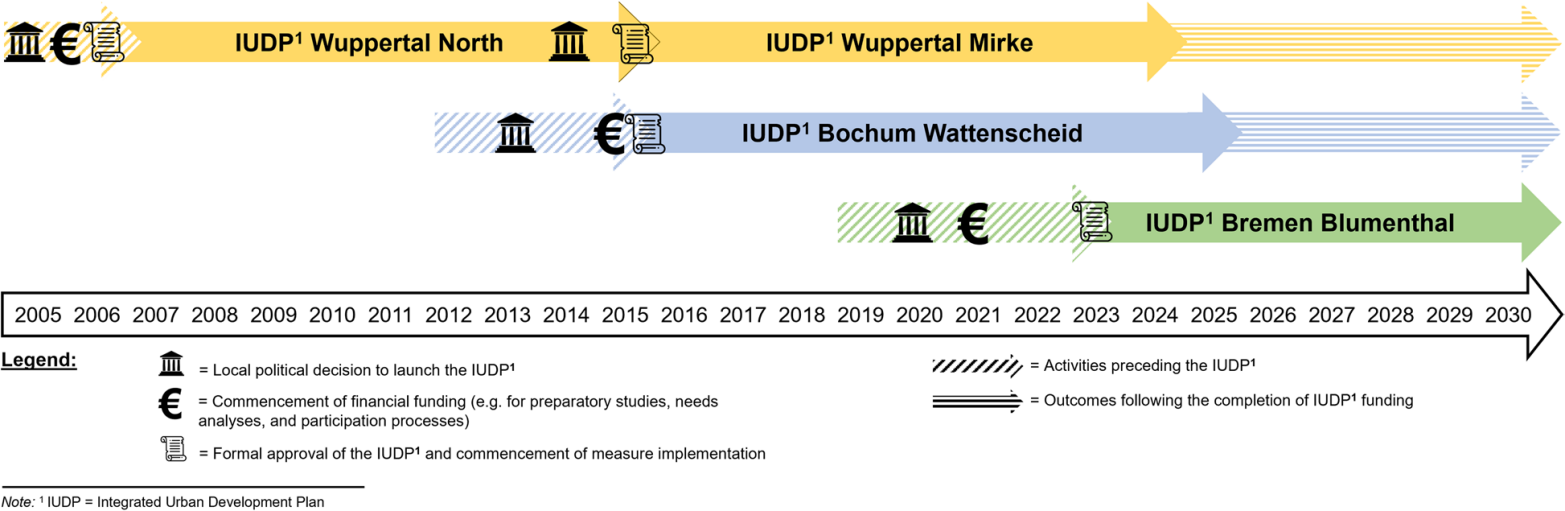
Timeline of the three Integrated Urban Development Plans (IUDPs)

IUDPs, as complex interventions, are developed over a multi-year process, adopted by municipal political committees, and then gradually refined and implemented over several years. This extended development and implementation phase lasts often six years or more. For SalusTransform, we selected three IUDP areas that differ in terms of their stage of IUDP implementation to be able to capture various aspects of implementation, outputs, outcomes and impacts (see Fig. 2).

In I1, the IUDP was approved by the Senate in April 2023, and the first measures were implemented at the end of 2023.

In I2, the implementation of IUDP measures, financed through the initial urban development funding, began in 2015. The second phase of urban development funding was commenced in early 2025, providing further financial resources for the implementation of additional measures.

In I3, the implementation of measures from a preceding IUDP began as early as 2007. With the update to the current extension in 2014, additional funding was made available, allowing further development of the area. The final measures will have been funded in 2026.

### Primary data collection methods

Following a mixed-methods approach, we will employ multiple data collection strategies, including: (1) two cross-sectional surveys conducted in the six study areas; (2) qualitative interviews with representatives of the authorities responsible for the formal implementation of the IUDPs, as well as key local stakeholders involved in on-site intervention delivery; (3) focus groups with residents of the intervention areas; (4) assessments of physical activity and sleep using accelerometers; (5) participatory formats involving the residents in the interventions areas, and (6) environmental assessments, including on-site visits, observations of active mobility and urban green spaces, as well as air-quality measurements.

#### Surveys of the residents

We will conduct two surveys in the study areas, one in the first and the other in the third year of the project. The surveys will target residents aged 18 years and older living within the defined study areas. In Bremen and Wuppertal, all residents will receive a personal access code enabling them to complete the survey online. In Wattenscheid-Mitte, due to the substantially larger population, a random sample of residents will be invited to participate, with the number of invitations adjusted to achieve a sample size comparable to the other intervention areas. The survey will be implemented on LimeSurvey, an open-source online survey tool [19]. The survey will include a set of validated questionnaires: Warwick Edinburgh mental well-being scale [20], Short Scale Measuring Perceived Social Participation [21], Oslo-3-Items-Social-Support Scale [22], self-efficacy short scale [23], and Abbreviated Place Attachment Scale (APAS) [24]. We will also use items on general mental and physical health status from the German Federal Health Reporting surveys, provided by the Robert Koch Institute (RKI) [25]. Furthermore, we will collect information about the participants’ living and housing situation, their subjective assessment of their living environment concerning (1) traffic infrastructure, (2) impairments due to noise, air pollution, and other environmental threads, (3) the proximity and accessibility of public green spaces, as well as their perceived impact on and involvement in urban planning decisions affecting their living environment. We will provide translations of the questionnaire in the most common languages of the study areas.

#### Interviews with stakeholders

To integrate the perspective of those responsible for and involved in the implementation of the IUDPs, we will conduct semi-structured interviews with local stakeholders. Interviewees will include both stakeholders responsible for implementing the IUDP and stakeholders who are either directly involved in implementing the measures on-site or are actively engaged in the development of the district in other ways. Due to the different stages of the IUDP processes in the three intervention areas, the interviews will take place at different times across the study sites: In Wuppertal, interviews will be conducted in the first, following those in Bochum in the second and in Bremen in the third project year.

For the recruitment process, a list of key stakeholders will be compiled. If additional relevant stakeholders are identified during the interview phase, these will be added to the interviews. Individuals will be contacted via email and provided with study information outlining the project’s purpose as well as objectives and data protection procedures. Prior to participation in the interview, all participants will be required to provide written informed consent.

The qualitative interviews address success factors and barriers of IUDPs with regard to ecological sustainability, social equity, and health. A semi-structured interview guide will serve as the basis for the interviews, organised chronologically according to the phases of the IUDP. The guide can be adjusted after each interview in terms of refining questions that have proven difficult or redundant.

Interviews will be conducted either in person or online and will be recorded. In case of online interviews, ZOOM will be used for both conducting and recording the sessions. The recordings will be transcribed automatically using the software noScribe (for more information: https://imt.uni-paderborn.de/software/noscribe).

#### Focus groups with residents

The perspective of the residents, as main target group of the IUDPs, is crucial for evaluating the impacts and processes. We will conduct twelve focus groups, four in each intervention area, to explore how the residents perceive and assess the IUDP process [26]. Participants will be purposively recruited from population groups that are considered particularly vulnerable due to living in relative poverty and are often overlooked in qualitative data collection. Each focus group will address the perspective of one of the following groups: adolescents, elderly, migrants from specific origin countries and communities, and people with disabilities. The focus groups will be conducted in the second year of the project in all three intervention areas.

#### Accelerometery

During both surveys, participants from I1 and C1 will be asked to participate in an accelerometer assessment on physical activity and sleep. Both will be assessed using an ActiGraph wGT3X-BT accelerometer (ActiGraph LLC, Pensacola, FL, USA), worn by participants on the non-dominant wrist 24 h for seven consecutive days. The device measures acceleration and deceleration of the body in three axes [27]. Sampling frequency will be set to 30 Hz. For downloading and pre-processing the measurement data, ActiLife (Version 6.15.0 ActiGraph LLC, Pensacola, FL, USA) will be used. Wear time periods will be determined based on the algorithm by Troiano et al. [28], with 60 consecutive minutes of zero counts defining non-wear time. For sleep, the Cole-Kripke scoring algorithm will be used to identify sleep and wake epochs [29]. Planned indicators are average daily counts per minute, daily minutes in moderate-to-vigorous physical activity (MVPA), total sleep time, sleep efficiency, and wake after sleep onset (WASO).

#### Participatory formats

To ensure meaningful integration of the residents’ perspectives into the evaluation process, we will actively involve them in different participatory formats. Residents will be directly approached and invited to take part in small-group activities that combine observation, discussion, and reflection. Examples include guided neighbourhood walks, during which participants explore their surroundings together and discuss ongoing changes, challenges, and potentials within the urban development process. During these neighbourhood walks, we will use photovoice to document participants’ gaze, capturing what draws their attention in the neighbourhood [30]. We will encourage them to take photos to document their lived experience, helping to reflect on and communicate what matters most to them in their local environment. In addition, meetings will be held at specific sites where IUDP interventions are planned, currently underway, or already completed. These discussions will focus on how residents perceive and use these spaces, what expectations and needs they associate with them, and how the interventions affect their everyday life.

#### Assessment of urban green

Based on Bläser et al. [31] and the German Federal Agency for Nature Conservation [32], we will assess urban green spaces with regard to quality of life and neighbourhood liveability, and examine how the IUDP contributes to socio-ecological transformation. With a focus on flora, the assessment applies specific criteria addressing the quality of flora within this transformation context. This includes mapping ecological indicators such as key plant species, invasive versus native species, and species of particular conservation concern – where possible fauna will be included. Data will be collected along representative circular walking routes that include comparable urban features (e.g., schools, playgrounds, parks) and intervention areas, sites affected by IUDP measures. The approach enables a systematic comparison of urban green quality and its relevance for socio-ecological transformation across all study areas.

#### Site visits to evaluate traffic infrastructure

By conducting standardised site visits and observations (based on Schnell et al. [33]), we aim to assess neighbourhood conditions for active mobility. Site visits will serve to develop a comprehensive understanding of the study area and its spatial as well as social characteristics [34]. They integrate various techniques, such as observation and mapping, and are particularly well suited or identifying local conditions in a structured manner. Two trained members of the project team – one familiar with the area and one without prior local knowledge – will carry out the observations to ensure diverse perspectives. The observers will document selected aspects such as infrastructure quality, spatial experience, environmental factors, safety, and accessibility using structured notes, photographs, and maps.

#### Air quality measurement

We will conduct exploratory measurements of particulate matter (PM2.5 and PM10) concentrations in all six study areas [35]. Data collection will begin in the first project year and continue at regular intervals until the conclusion of the project. Currently, only data from official monitoring stations along major roads are available, which provides limited insights into air quality variation within neighbourhoods. In each neighbourhood, three measurement sites – representing low, medium, and high expected pollution levels – will be identified using a Geographic Information System (GIS). This approach enables the analysis of spatial factors such as population density, land use, and traffic intensity. Measurements will be carried out using Senseboxes [36], low-cost sensors that record particulate matter in real time. Observers will document PM concentrations along with GPS coordinates, measurement time, wind speed, and key site characteristics, including proximity to roads or green spaces. To maximise comparability, the short-term measurements will be conducted on weekdays under similar weather conditions.

### Secondary data sources

In addition to primary data, we will collect secondary data from various sources provided by the respective city authorities. These data will include information on the population structure of the study areas (e.g. number of inhabitants, age distribution, proportion of residents with a migration background, unemployment rates, and other sociodemographic indicators), as well as aggregated health and environmental data. Health data will include indicators such as life expectancy, results from school entry examinations, and other aggregated public health statistics. Environmental data will include information on air and noise pollution, heat exposure, and the availability and quality of green spaces.

For the intervention areas, we will also gather detailed information on the IUDPs’ implementation processes from the responsible authorities, including data on expenditures, participation rates, and the use of specific interventions such as counselling services or small subsidy programmes for private homeowners. Additionally, we will document any delays in the implementation and their underlying causes. To ensure a longitudinal perspective, secondary data will be collected retrospectively for a period of ten years prior to the start of the respective IUDP and continuously throughout the project duration.

In the control areas, we will assess whether any interventions are planned or implemented that target similar dimensions as the IUDPs but have not been formally developed or approved as such. Close cooperation with local authorities and the wider network of practitioners in the study areas, established during the project conception phase, will ensure comprehensive and reliable access to these data sources.

### Eligibility criteria

To participate in primary data collection activities, residents must be at least 18 years old, and their main residence must be within the respective study area. For the focus groups, we will consider including residents aged 16 and older to capture the perspectives of younger population groups.

### Recruitment of the participants

For the surveys, contact details of potential participants will be provided by the local residents' registration office. These contact details will be stored in a secure database on a server at the University of Bremen. We will send an invitation letter to all potential participants, which includes study information and an individual code to access the online survey. Informed consent will be obtained immediately before the survey begins.

At the end of the survey, participants will be asked whether they are interested in taking part in an additional assessment using accelerometers to measure physical activity and sleep. The accelerometer assessment will be described and an info sheet with more details will be attached for download. Interested individuals can contact the study team via a dedicated phone number or email address to receive further information and to schedule the assessment period. During an in-person appointment at the participant’s home, written informed consent will be obtained, and the accelerometers will be handed out.

For the interviews with stakeholders, the focus groups with residents, and further participatory formats, participants will be recruited through personal contact facilitated by cooperating stakeholders.

Throughout the entire project duration, we will engage in promotional activities for both the overall project and for specific data collections (such as surveys or interviews) to support recruitment and increase public awareness of the project. For these purposes, a project website and various social media accounts have been created. Face-to-face engagement and dialogue will also be supported through participation in local events, such as health promotion days, summer fairs, family festivals, neighbourhood council meetings, and local working group sessions related to urban development. In addition, we contacted local newspapers to publish tailored information about the project and the data collections relevant to each study area.

### Allocation

The allocation to either an intervention area or a control area is defined by the participants’ main residence.

### Interventions

The IUDPs comprise a broad variety of interventions. In the following, we describe the different interventions of the respective IUDP.

#### IUDP 1 (Bremen)

In Bremen Blumenthal, the IUDP was launched in 2023 with the aim of revitalising the historic district and creating healthier, more equitable living environments. The programme combines spatial, social, and physical environmental interventions to address long-standing challenges of structural inequality and urban neglect.

A significant contextual development is the construction of a new vocational training campus on the site of the former local wool factory, a large-scale industrial brownfield site. While formally not part of the IUDP, the campus is expected to attract thousands of students and professionals, offering new opportunities for education and employment as well as reshaping the district’s social and economic dynamics. The IUDP builds on this transformation by ensuring that the campus is well integrated into local life – for example through new pedestrian links to the district centre and measures that facilitate broad and inclusive access to its opportunities.

Complementary interventions within the IUDP focus on strengthening the social and cultural infrastructure of the historic centre. The former town hall will be renovated and repurposed as a multifunctional neighbourhood hub, hosting a library, community spaces, and social services to provide low-threshold access to resources and support. The surrounding market square, adjacent streets, and a large public green space will be redesigned to become more attractive, accessible, and inclusive areas for recreation and encounter, encouraging greater use by diverse groups of residents.

To counter widespread vacancy and housing neglect, the programme supports property owners with funding for renovations, energy-efficient upgrades, and heritage-sensitive restoration. In addition, the municipality has acquired several dilapidated housing units for renovation and reallocation as socially managed housing. These measures aim to ensure decent living conditions, reduce housing-related health risks, and prevent segregation and displacement.

The IUDP integrates a broad range of climate mitigation and adaptation measures. By preserving and upgrading existing buildings, the programme aims to retain “grey energy” while improving energy efficiency and exploring alternative energy systems. Renovations will favour low-carbon materials where possible, while adaptation strategies include minimising surface sealing, promoting nature-based rainwater management, and introducing green roofs or façades. Further elements such as shading contribute to urban cooling, reduced heat stress, and improved well-being. Collectively, these measures are expected to contribute to the creation of healthy, climate-resilient, and accessible living environments for diverse members of the community reflecting an integrated approach to urban sustainability.

#### IUDP 2 (Bochum)

In Bochum Wattenscheid-Mitte, the IUDP was launched in 2015 with the aim of revitalising the inner-city area of Wattenscheid, a former independent city that became part of Bochum following local government reorganisation in 1975. Wattenscheid was shaped by an active coal mining industry until the mid-1970s and due to structural economic changes is characterised today by a comparatively disadvantaged population. Children in particular show poorer health outcomes at school entry examinations than in other districts of the city, including higher rates of overweight, lower motor skills, and more frequent hearing impairments.

The overall objective of the IUDP is to create a healthier and more family-friendly neighbourhood. The programme combines spatial, social, and physical environmental interventions to address long-standing structural inequalities, with a particular focus on deficits in urban planning and the built environment. Since 2015, several renewal interventions have been completed, including the redesign of parks such as the Friedenspark am Ehrenmal and the Stadtgarten, which now offer diverse functions for play, recreation, and culture. The redesign of Monte-Schlacko – a park located on a former mine dump – is currently underway. Another example of ongoing spatial transformation is the conversion of a former sports field into a multifunctional space for wheel-based active mobility, complemented by tree planting and a rainwater retention basin as part of climate adaptation strategies.

Other interventions include the redesign of playgrounds and schoolyards, as well as the redevelopment of the central square. While the square is both the neighbourhood’s main public transport hub and heavily sealed, making it a recognised heat hotspot in summer, it has long been perceived as lacking comfort and urban quality. Its redevelopment therefore aims to improve both usability and climate resilience, creating a more attractive and comfortable public space for residents. Complementary schemes such as façade improvement grants encourage private investment in the upgrading of residential buildings.

Community funding within the IUDP framework supports a wide range of social and health-related projects. For instance, the annual Health Weeks held each spring provide health-related activities for residents and facilitate networking among local stakeholders.

In line with the guiding principle of health and family, considerable efforts are made to promote health besides improving the built environment. For more than ten years, the local neighbourhood management has been building a health network that connects different actors in health promotion and healthcare. One major achievement is the opening of a Community Health Centre (“health kiosk”) in spring 2025, after years of preparation. The centre aims to guide diverse communities through the German health system, improve health literacy, and support the development of new health services. It is co-financed by a health insurance fund and the City of Bochum, operated by a dedicated company that includes a local charity organisation.

The IUDP also acts as a platform to mobilise additional resources and partnerships. Between 2020 and 2024, a joint project of the city’s planning and health departments, neighbourhood management, a health insurance fund, and a local university promoted physical activity on redesigned public sites and developed tailored outreach activities for groups that are often hard to reach. Among these, a cycling course for migrant women offered by the Bochum city sports association illustrates how targeted, low-threshold offers can contribute to social inclusion and health promotion at the neighbourhood level.

#### IUDP 3 (Wuppertal)

In Wuppertal, two successive IUDPs have been implemented, with the first being launched in 2007 and later extended and renewed in 2014, reflecting a long-term, evolving strategy for urban development.

The first IUDP (2007) was based on an integrated approach aiming to combine structural upgrades, improved infrastructure, and social stabilisation. The overarching goal was to develop Elberfeld's northern district (a former independent city, incorporated into Wuppertal in 1929 and formalised under the 1975 local government reorganisation), including the Ölberg and Mirke areas, into an attractive, liveable, and sustainable district where urban quality, social inclusion, and community engagement go hand in hand.

Particular focus was placed on strengthening the high-quality residential areas in the northern district, especially the historicist buildings from the “Gründerzeit” period (late nineteenth century), and on creating new green spaces and playgrounds, addressing a previous lack of attractive recreational areas. The aim was to improve quality of life and prevent families from leaving by upgrading existing facilities. At the same time, a balanced land-use plan had to be developed, as the creation of additional green spaces competed with the high demand for parking. These efforts sought to enhance both quality of life in the neighbourhood and transport infrastructure for residents and local businesses. Social integration and educational support were equally important, as Elberfeld's northern district continues to be characterised by a high proportion of socially disadvantaged households despite positive developments. Expanding educational opportunities, promoting equal opportunities, and strengthening social cohesion were considered crucial for maintaining the district’s predominantly positive image over the long term.

Following the initial 2007 IUDP, the 2014 IUDP focused on maintaining or restoring the attractiveness of the districts as lively neighbourhoods with a mix of residential and commercial properties, while preventing further social and functional segregation. The programme focused on improving the living environment through new uses and repurposing of land and buildings, attractive façade design, developing concepts for commercial brownfield sites, refurbishing industrial monuments and backyards for new uses, and revitalising vacant shop premises. Throughout the framework of IUDP, issues such as gender mainstreaming, sustainability, demographic change, integration of people with a migration background, and inclusion of people with impairments and disabilities were consistently prioritised and embedded in both objectives and measures.

The central development goals of the IUDP included establishing Utopiastadt (literally “City of Utopia,” a community-driven urban development and cultural project) in the historic Mirke railway station as a laboratory and centre of excellence for urban, cultural, and social development, thereby reactivating the disused railway site as a business park, strengthening sustainable mobility, developing urban gardening as both an ecological and social strategy, and generally positioning the Mirke district as an intercultural hotspot. In addition, the programme aimed to develop the “Gründerzeit” buildings into an attractive residential area, promote zero-energy and energy self-sufficiency, and create model housing for older people with a migrant background.

### Outcomes

**Fig. 3:**
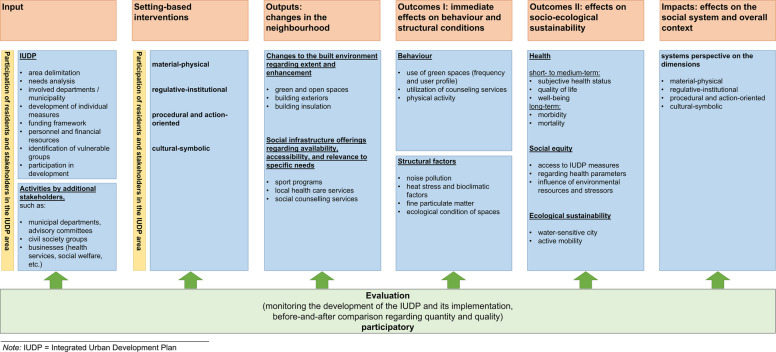
Logic model of the evaluation framework for Integrated Urban Development Plans

We will evaluate the IUDPs’ effects in relation to three entangled key dimensions: residents’ health, social equity, and ecological sustainability. To capture the complexity of change processes, we distinguish between different analytical levels, reflecting short- to long-term developments: “Outputs” refers to directly observable, short-term changes in the neighbourhood’s physical and social environment (e.g. new infrastructure, renovated public spaces), “Outcomes I” relates to early behavioural changes and modifications in local structural conditions, “Outcomes II” addresses medium-term effects on socio-ecological sustainability, such as increased participation or improved environmental quality, and “Impacts” describes long-term effects and more fundamental changes at the level of the broader societal system and overall context, such as strengthened resilience or reduced inequalities. To support this evaluation framework, we have developed a generic logic model applicable to all three IUDPs illustrating these levels and their interconnections (see Fig. 3).

#### Residents’ health

Mainly, we will include subjective health status and different aspects of mental health to describe changes in the residents’ health. Health-related quality of life will be assessed using items from the German Federal Health Reporting surveys by the RKI, which include questions on general health status as well as physical limitations affecting daily activities. To complement this, we will measure the residents’ well-being, focusing on the more subjective and emotional aspects of their living conditions [20]. Additionally, the social network and integration will be evaluated by assessing the residents' social support [22], specifically the number of close people providing them with help and support.

#### Social equity

Regarding social equity, we will examine different aspects of environmental justice, namely distributional and procedural justice [37]. Distributional justice will be operationalised by analysing social disparities in exposure to environmental stressors as well as access to resources. Using survey data, we will compare living and housing conditions across different population groups, stratified by various socioeconomic factors such as income, education, migration experience, and employment status. Additionally, survey data will be used to analyse which social groups will be affected – positively or negatively – by potential changes resulting from the IUDP interventions.

To assess procedural justice, we will explore participants’ involvement in environmental and political decision-making. We will distinguish between three aspects: their knowledge of opportunities to participate in decision-making processes, their intention to engage – such as by signing a petition, attending an information event, or submitting a complaint to local authorities – and their actual past participation.

We will also use the Short Scale Measuring Perceived Social Participation to capture participants' perspectives on how they perceive their role within society [21].

#### Ecological sustainability

To assess ecological sustainability, we will draw on multiple data sources. Information on urban green spaces relevant to quality of life and neighbourhood liveability will be collected through on-site inspections conducted by members of the study team. Complementary secondary data on the number and condition of trees within the study areas will be obtained from the respective local authorities. Additional secondary data from these authorities will include information on the proportion of sealed surfaces, which serves as an indicator of the area’s infiltration capacity.

With regard to sustainable mobility, we will assess the share of active mobility in the study areas based on participants’ self-reported usual modes of transport in the survey. Furthermore, we will provide an overview of the neighbourhood conditions that support or hinder active mobility.

### Participant timeline

Due to the mixed-methods approach, the study does not follow a single, linear participant timeline but instead comprises a complex structure of multiple data collections and assessments. These involve different participant groups and vary across study areas with respect to both timing and content. This design allows for the integration of complementary perspectives and the examination of change processes at different levels and stages, thereby providing a more comprehensive understanding of the health, social, and ecological impacts of the IUDPs.

### Data analyses

To evaluate the effectiveness of IUDPs as complex interventions, the intervention areas will be compared with the control areas in regard to the aforementioned evaluation parameters. Regarding the IUDPs’ implementation, this includes an analysis of specific outputs, such as structural or socio-spatial changes in the neighbourhoods. In addition to the assessment of these outputs, we will focus on different outcome parameters in each city: in Bremen (I1, C1), these include Outcomes I and short-term Outcomes II; in Bochum (I2, C2), short- and long-term Outcomes II; and in Wuppertal (I3, C3), long-term Outcomes II and Impacts (Fig. 3).

Both bivariate and multivariate analyses will be conducted using established statistical procedures appropriate to the data structure. Quantitative data from the cross-sectional surveys at two time points, environmental data emerged from the different data collection formats, as well as secondary quantitative data collected at multiple time points, will be analysed using a difference-in-differences (DiD) approach [38]. Both absolute (difference-in-differences) and relative changes (ratio-of-ratios) will be assessed, and inequality effects will be examined through stratification by various social indicators [39]. From an intersectional perspective, social indicators will be selected based on the PROGRESS-Plus framework, as recommended by the Campbell and Cochrane Equity Methods Group [40]. The quantitative data analysis will be carried out using SAS (version 9.4 or higher) and R (version 4.5.1 or higher) [41].

Qualitative data from semi-structured interviews and focus groups will be analysed using the method of qualitative content analysis as described by Kuckartz and Rädiker [42]. Unlike Mayring’s approach [43], this method allows not only category-based but also case-based analysis. For the present study, the content-structuring variant of content analysis will be applied [42]. The aim is to systematically structure the material by content. Data analysis will be conducted using MAXQDA software. To ensure quality of the coding process, the recommendations of Kuckartz and Rädiker [42] regarding intercoder agreement will be followed. Previous to the initial coding round, the main categories will be jointly defined by the study team and the interviews will then be independently coded by two team members using these main categories. During this process, the coders will record open questions and ideas for modifying the code tree. Afterwards, the two team members will compare their codes, agree on a consolidated version, and document this in the codebook. In addition, the identification of themes and subthemes will be discussed and agreed upon within the entire study team.

Accelerometer data will be processed using ActiLife software (ActiGraph, Pensacola, FL). Non-wear times will be defined based on the Troiano algorithm [28]. Acceleration measurements across three axes will be aggregated into vector magnitudes and expressed as average counts per minute. Minimum wear time is defined as 22 h per day on at least five consecutive days. Sleep scoring will be conducted according to algorithms developed by Cole and Kripke [29].

Access to study data will be restricted to members of the study team, and data storage will comply with applicable legal regulations.

### Monitoring

To monitor the study, a Steering Committee (SC) and a Data Monitoring Committee (DMC) will be established. The SC will include all researchers involved in this publication and will be chaired by GB, an experienced public health researcher with expertise in interdisciplinary research on health equity and environmental justice. In regular meetings, the SC will oversee study procedures to ensure adherence to the study protocol. Key areas of focus will include recruitment processes across all data collection formats, data collection procedures, and communication with local stakeholders. The primary aim of the SC is to facilitate the smooth conduct of the study.

The DMC will consist of five members, including one data manager from the Department of Social Epidemiology at the Institute for Public Health and Nursing Research, University of Bremen. Members will possess epidemiological, statistical, and research expertise. The DMC will regularly monitor data quality and plausibility by reviewing exports from the online survey tool. Since we will not implement any intervention by ourselves, no interim analyses or auditing are planned.

## Discussion

In SalusTransform, IUDPs will be evaluated for the first time in a comprehensive manner with regard to their real-world effects on residents’ health, social equity, and ecological sustainability. Drawing on diverse data sources from three different cities in Germany, we aim to gain an in-depth understanding of how IUDP measures have been implemented and what impacts they have on the living conditions of residents in the intervention areas and on the extent of environmental health inequities compared to similarly structured control areas within the same cities.

One major challenge we anticipate is ensuring participant retention across the various data collection formats. To address this, we will implement tailored strategies to facilitate participation. During the survey phases, we will attend local events in all study areas to promote the online surveys and engage directly with residents. Additionally, we will offer on-site support on selected dates to assist participants who experience difficulties completing the survey or need help reactivating their single-use access code. These support activities will be communicated through the project website, the project’s social media channels, and a general study hotline that will be available during weekdays.

Another challenge may occur when it comes to significant delays in timing and extent of the implementation of IUDP measures in Bochum and Bremen. To evaluate the process of implementation, we will collect information on reasons for any delays and potential countermeasures. Moreover, our evaluation approach focuses on the bundle of measures put into practice instead of analysing only selected single measures.

Finally, developments within the control areas that lead to the implementation of interventions comparable to an IUDP, or even to the initiation of an IUDP planning process, may pose an additional challenge. We will address this by closely monitoring developments in the control areas and applying a difference-in-differences analysis. A strength of our evaluation approach is the observation of control areas specifically identified and assigned to the IUDP area in each of the three cities. These control areas face similar challenges concerning population characteristics, infrastructure and environmental conditions, but are not located directly adjacent to the intervention areas, thus reducing the risk of spill-over effects of IUDP activities on the control areas.

The results of this study will be presented to academic, municipal, and public audiences. During the project period, city-specific information tailored to residents, practitioners, and local policy representatives will be disseminated via the project website and local events such as neighbourhood or community meetings. Final dissemination events in each city will present key results and discuss their implications for health-promoting, socially equitable, and environmentally sustainable urban development. Continuous collaboration with municipal stakeholders will support transdisciplinary exchange and contribute to the institutionalisation of evaluation practices for urban renewal measures. Scientific dissemination will include publications in peer-reviewed journals and presentations at national and international conferences.

In the longer term, the project seeks to transfer its evaluation approach to other municipalities through established research and practice networks. Furthermore, results and methodological insights will be integrated into teaching and professional training at the three participating research institutes.

This study was prospectively registered with the German Clinical Trials Register (DRKS00036042) on May 28th, 2025.

## Supplementary Information

Supplementary Material 1.

## Data Availability

No datasets were generated or analysed during the current study.
